# Targeting NFAT2 for Reversing the P-gp-Mediated Multidrug Resistance to Paclitaxel by Manidipine

**DOI:** 10.3390/cancers17203289

**Published:** 2025-10-10

**Authors:** Jian Zhou, Nan Wang, Yu-Kang Lin, Qi-Lu Li, Rui-Ming Liu, Jia-Qin Hu, Hua Zhou, Hai Lan, Ying Xie

**Affiliations:** 1 State Key Laboratory of Traditional Chinese Medicine Syndrome, Chinese Medicine Guangdong Laboratory (Hengqin Laboratory), The Second Affiliated Hospital of Guangzhou University of Chinese Medicine, Guangzhou 510006, Chinalql20001216@163.com (Q.-L.L.); gutcmzhs@hotmail.com (H.Z.); 2 State Key Laboratory of Quality Research in Chinese Medicines, Macau University of Science and Technology, Macau SAR, China; 3 Shunde Affiliated Hospital to Guangzhou University of Chinese Medicine, Guangzhou 528300, China

**Keywords:** MDR, P-gp, manidipine, NFAT2, calcium signaling

## Abstract

Background: Multidrug resistance (MDR), primarily driven by P-glycoprotein (P-gp)-mediated drug efflux, presents a significant challenge in cancer therapy, contributing to chemotherapy failure and poor patient outcomes. Objectives: In this study, we explored the potential of manidipine (MA), a clinically approved calcium channel blocker, to reverse P-gp-mediated MDR through modulation of calcium signaling via nuclear factor of activated T cells 2 (NFAT2). Methods: Paclitaxel (PTX) resistance ABCB1-overexpressing cancer in vitro and in vivo were used for evualting the anti-MDR effects of MA, as well as the underlying mechanism with siRNA of NFAT2. Results: We found that MA at non-toxic concentrations (0.6–5.4 μM) significantly sensitize drug-resistant colorectal (HCT-8/T) and non-small cell lung (A549/T) cells to PTX, reducing its IC_50_ by up to 1328-fold in vitro models. Mechanistically, MA inhibited P-gp efflux activity without altering its expression, as shown by an increased intracellular accumulation of doxorubicin and Flutax-2 (2.3- and 3.1-fold, respectively) and dose-dependent modulation of ATPase activity (EC_50_ = 4.16 μM). Notably, MA reduced intracellular calcium levels (52% reduction, *p* < 0.001) and downregulated NFAT2, an oncogene overexpressed in resistant cells. In vivo, MA (3.5 mg/kg) synergizes with PTX to inhibit tumor growth by 68% (*p* < 0.001) in A549/T xenograft model, without an observable decrease in weight. Conclusions: In sum, all these results position MA as a novel NFAT2 inhibitor to overcome P-gp-mediated MDR via modulating calcium signaling, which points to further investigation for its clinical applications.

## Simple Summary

Multidrug resistance (MDR) is a major problem in cancer treatment, often caused by a protein called P-glycoprotein (P-gp) that pumps chemotherapy drugs out of cancer cells. This makes treatments less effective and leads to poor outcomes for patients. In this study, we investigated manidipine (MA), a drug used to treat high blood pressure, to see if it could reverse this resistance. We found that MA, at safe doses, can make drug-resistant cancer cells more sensitive to chemotherapy drugs like paclitaxel. It does this by changing calcium levels inside cells and reducing the activity of a protein called NFAT2, which is linked to resistance. In experiments, MA significantly improved the effectiveness of chemotherapy and reduced tumor growth in mice, without causing harmful side effects. Our findings suggest that MA could be a promising new approach to overcoming drug resistance in cancer treatment.

## 1. Introduction

Multidrug resistance (MDR) remains a significant challenge in cancer treatment, responsible for more than 90% of chemotherapy failures and a substantial proportion of cancer-related deaths [1,2]. While chemotherapeutic agents such as paclitaxel (PTX) and doxorubicin (DOX) serve as frontline treatments for advanced malignancies, their long-term efficacy is frequently compromised by acquired drug resistance. Efforts to counteract multidrug resistance have focused on agents that either lower ABC-protein levels or block their efflux activity. Yet the first-, second-, and third-generation modulators—including quinine, verapamil, tariquidar, LY335979, and GF120918—achieved reversal only at high doses, which were accompanied by unacceptable toxicities [3,4]. Over the past two decades, despite extensive identification of human MDR pump modulators, fewer than 1% of these compounds have progressed to clinical evaluation [5], underscoring an urgent need for safer, more effective resistance modulators targeting efflux mechanisms without systemic toxicity.

The pathobiology of MDR involves multifactorial mechanisms [4], including enhanced drug efflux through ATP-binding cassette (ABC) transporters, impaired drug uptake, dysregulated DNA repair processes, and drug target mutations [6,7]. Among these, the overexpression of P-glycoprotein (P-gp), a key member of the ABC transporter family, is a predominant factor in conferring resistance by actively expelling chemotherapeutic agents, thereby reducing their intracellular concentration and efficacy [8]. Targeting P-gp has been a primary focus in overcoming MDR [9,10]. Conventional P-gp inhibitors, such as verapamil, have been demonstrated to be effective in sensitizing cancer cells to chemotherapy. However, their clinical utility is limited by high toxicity and off-target effects [11,12]. These challenges have driven the search for safer, more effective compounds capable of modulating P-gp function.

The P-gp-inhibitory effect of thousands of compounds was calculated by molecular docking simulations in the primary study. The analyses revealed that Manidipine (MA), a dihydropyridine calcium channel blocker approved for hypertension management [13,14,15], have strong bond interactions with P-gp. MA has shown anti-inflammatory [16] and nephroprotective effects [17] in previous studies. Moreover, calcium signaling pathways play an important role in acquired drug resistance [18,19] by modulating gene or protein expression in multidrug-resistant cancer cells, such as Sorcin [20], SERCA [21], and TRP channels [22].

Recently, high expression of the nuclear factor of activated T cells (NFAT) family, which is primarily activated by store-operated calcium entry, acts as a critical modulator in cancer progression [23] and drug resistance [24], such as NFAT nuclear translocation inhibitor Bcl-2 [25] and cyclosporine A [26].

Therefore, the use of an NFAT inhibitor in combination with anti-cancer drugs is a promising approach to overcoming cancer drug resistance. However, which NFAT isoform could be a suitable target in PTX-related MDR remains unclear, and there are currently no NFAT inhibitors on the market. Thus, there is still an urgent need to develop effective NFAT inhibitors that can produce therapeutic effects.

Building on these insights, this study aims to evaluate the role of manidipine in reversing P-gp-mediated MDR by exploring its effects on calcium signaling via NFAT2 activity to elucidate the molecular mechanisms underlying its potential to enhance chemotherapeutic efficacy and provide a foundation for future clinical applications in MDR cancer therapy.

## 2. Materials and Methods

### 2.1. Reagents and Cell Culture

Manidipine dihydrochloride (MB5034), paclitaxel (MB1178), docetaxel (TXT, MB1081), doxorubicin (DOX, MB1087), verapamil (Vera, MB1346), 5-fluorouracil (5-FU, MB1273), and daunorubicin (DAU, MB1074) were purchased from Dalian Meilun Biotechnology Co., Ltd. (Dalian, China) and stored at −20 °C. Fluorescent probes 4’,6-diamidino-2-phenylindole (DAPI, D9542) and propidium iodide (PI, 537059) were obtained from Sigma-Aldrich (St. Louis, MO, USA). Flutax-2 (Flu-Tax, P22310), fetal bovine serum (1009148), penicillin-streptomycin-glutamine (10378016), RPMI 1640 medium (C11875500), and streptomycin were sourced from Life Technologies Inc. (Grand Island, NY, USA). Ham’s F-12K medium (PM150910) was procured from Procell Co., Ltd. (Wuhan, China).

Fluo-4 AM (S1060) was purchased from Beyotime Biotechnology Co., Ltd. (Xiamen, China). Cyclosporin A (CsA, HY-B0579) was acquired from MedChemExpress Co., Ltd. (Monmouth Junction, NJ, USA). Calcium chloride (R00447) was obtained from Beijing Leagene Biotechnology Co., Ltd. (Beijing, China). Antibodies against P-gp (13342S) and GAPDH (BA2913) were purchased from Cell Signaling Technology, Inc. (Danvers, MA, USA) and Boster Co., Ltd. (Pleasanton, CA, USA), respectively. NFAT2 (66963-1-Ig) and calmodulin (10541-1-AP) antibodies were purchased from Proteintech Co., Ltd. (Rosemont, IL, USA). NFAT2-targeting siRNA oligos and Lipofectamine 3000 (L3000-015) were procured from Generay Co., Ltd. (Seongnam-si, Republic of Korea) and Invitrogen Co., Ltd. (Carlsbad, CA, USA), respectively.

Human colorectal adenocarcinoma HCT-8 cells and their PTX-selected ABCB1-overexpressing resistant subline (HCT-8/T) were kindly provided by Prof. Zhi-Hong Jiang (Macau University of Science and Technology, Macau SAR, China). Human non-small cell lung cancer (NSCLC) A549 cells (KGG3215-1) and their resistant counterpart A549/T (CL-0585) were purchased from KeyGen Biotech Co., Ltd. (Nanjing, China) and Procell Co., Ltd. (Geneva, Switzerland), respectively. HCT-8, HCT-8/T, and A549 cells were cultured in RPMI-1640 medium, while A549/T cells were maintained in Ham’s F-12K medium, supplemented with 10% FBS and 1% penicillin-streptomycin at 37 °C under 5% CO_2_.

### 2.2. Cell Viability Assay

Cell viability was assessed using the sulforhodamine B (SRB) assay [27]. Briefly, cells (6 × 10^3^/well) were treated with manidipine (0.6, 1.8, or 5.4 μM) combined with varying PTX concentrations for 48 h. After fixation with 10% trichloroacetic acid, cells were stained with 0.4% SRB in 1% acetic acid. Bound dye was solubilized in 10 mM Tris buffer, and absorbance was measured at 515 nm using a microplate reader (Spectra MAX 250; Molecular Devices, San Jose, CA, USA). The MDR reversal fold was calculated as the ratio of IC_50_ values for anti-cancer drugs in the absence versus the presence of manidipine.

For the colony formation assays, HCT-8/T cells (1200 cells/well) were plated in 6-well plates, and after 24 h the cells were treated with 0.94 μM PTX or combine them with varying concentrations of MA for 8 days. Following the removal of the medium from the wells, cells were stained with 0.4% crystal violet at room temperature for 30 min. Cells were washed twice with PBS to remove excess dye. Images were captured and counted.

### 2.3. Flow Cytometric Analysis of Cell-Cycle Status and Apoptosis Analysis

Cell cycle analysis was assessed using flow cytometric. After treatment with drugs for 48 h, cells were collected and resuspended in ice-cold PBS, then fixed overnight in ice-cold 70% ethanol at 4 °C. Cells that had been fixed were removed from the refrigerator and equilibrated at room temperature for 15 min. After centrifugation, cells were washed twice with PBS, and propidium iodide (PI) staining solution (50 µg/mL PI and 200 µg/mL RNase A in PBS) was added and incubated for 15 min at room temperature in the dark. The samples were further examined by flow cytometry, BD FACS Aria III (BD Biosciences, San Jose, CA, USA).

For apoptosis analysis, after drug treatment for 48 h, 1 × 10^6^ HCT-8/T cells were collected and washed once with binding buffer. Apoptotic cells were re-suspended in 100 μL binding buffer containing 5 μL Annexin V-FITC and 5 μL propidium iodide (PI) (50 μg/mL) following incubation for 10 min at room temperature in the dark. After incubation, the mixture was analyzed by FACS using an Aria III flow cytometer (BD Biosciences, San Jose, CA, USA).

### 2.4. Drug Combination Assay

Drug interactions were evaluated using the Chou-Talalay method [28,29]. HCT-8/T cells (6 × 10^3^/well) were exposed to serial dilutions of PTX (IC_50_ = 4.16 μM) and manidipine (IC_50_ = 12.67 μM), alone or in combination, for 48 h. Cell viability was determined by SRB assay, and combination indices (CI) were calculated using CalcuSyn v2.1 (Biosoft, Novosibirsk, Russia). Synergy was classified as follows: CI < 0.1 (very strong synergism), 0.1–0.3 (strong synergism), and 0.3–0.7 (synergism).

### 2.5. P-gp ATPase Activity Assay

P-gp ATPase activity was measured using the Pgp-Glo™ Assay Kit (Promega, Madison, WI, USA) per manufacturer guidelines. Luminescence was quantified (infinite M200 PRO, TECAN, Männedorf, Switzerland) to assess manidipine’s inhibitory effects on verapamil-stimulated ABCB1 ATPase activity, with sodium orthovanadate (Na_3_VO_4_) as a negative control.

### 2.6. Intracellular Uptake and Accumulation Assay

For fluorescence microscopy, HCT-8 and HCT-8/T cells (1 × 10^5^) on coverslips were treated with DOX (5 μM) or Flu-Tax (2 μM) ± manidipine (5.4 μM) or verapamil (50 μM) for 4 h. Cells were fixed in 4 wt% formaldehyde (Sigma-Aldrich, St. Louis, MO, USA), nuclei stained with DAPI (1 μg/mL), and imaged (Leica DM2500, Leica, Wetzlar, Germany).

DOX accumulation in A549/T cells was quantified by flow cytometry. Cells were treated with DOX (1 μM) ± manidipine (5.4 μM), CsA (15 μM), or verapamil (50 μM) for 4 h, washed, resuspended in PBS, and analyzed (BD FACS Aria III, BD Biosciences, San Jose, CA, USA).

### 2.7. Animals and Xenograft Model

Experiments were carried out in accordance with the Guidelines on Animal Care and Experiments of Laboratory Animals (Center of Experimental Animals, Sun Yat-Sen University, China), which was approved by the ethics committee for animal experiments. A549/T cells (2 × 10^6^) were subcutaneously injected into the right flank of 4-week-old BALB/c nude mice (purchased from Sun Yat-Sen University, Guangzhou, China). When tumors reached ~100 mm^3^ (day 0), mice were randomized into six groups (n = 6): Saline solution (control); PTX (10 mg/kg); MA (3.5 mg/kg); PTX with high dose MA (10 mg/kg PTX and 3.5 mg/kg MA); PTX with low dose MA (10 mg/kg PTX and 1.75 mg/kg MA); PTX with Vera (10 mg/kg PTX and 10 mg/kg Vera). The vehicle used to deliver the MA and PTX were Cremophor EL/ethanol/saline (5%/5%/90%). The mice were treated via intraperitoneal injection (i.p.) every 2 days for a total of 9 doses. The animals were euthanized under isoflurane anesthesia at the end of the experiment as required. In addition, we followed the ARRIVE (Animal Research: Reporting of In Vivo Experiments) guidelines. Tumor volume (length × width^2^/2) and body weight were monitored. Tumors and organs were harvested post-euthanasia for analysis.

### 2.8. Calcium Content Assay Using Fluo-4 AM

In-cell measurement of calcium signaling was performed using Fluo-4 AM according to the manufacturer’s protocol. Fluo-4 AM is a fluorescent Ca^2+^ indicator, and its fluorescence is increased upon binding to Ca^2+^. Briefly, after treatment, medium from the culture plate was removed, and 200 µL of 2μM Fluo-4 AM dye loading solution was added to each well quickly. Cells were incubated at 37 °C for 30 min. The calcium response was measured by measuring the fluorescence using a 488 nm argon laser for excitation at 494 nm and emission at 516 nm.

### 2.9. Western Blot Analysis

After washing 3 times with ice-cold PBS, treated samples were lysed on ice for 30 min using ice-cold RIPA buffer. After sufficient lysis, the protein concentration of total protein lysates was measured using the Bicinchoninic Acid (BCA) assay and then added to the loading buffer and boiled for 5 min. Equal amounts of total protein lysates after SDS-PAGE separation were transferred to PVDF membranes. After sealing with 5% skim milk powder or 5% bovine serum albumin (BSA), the PVDF membranes were immunoblotted with primary antibody overnight at 4 °C. After incubation with the corresponding secondary antibodies at room temperature, protein expression was detected using an ECL Western blot analysis system

### 2.10. NFAT2 Knockdown

Lung cancer cells were seeded in 6-well plates and allowed to adhere at 37 °C for 24 h. siRNA oligos against NFAT2 (siNFAT2) and negative control (siNC) were delivered into lung cancer cells with Lipofectamine 3000 according to the manufacturer’s instructions, followed by incubation at 37 °C for 72 h.

The NFAT2 siRNA sequences are as follows. siRNA 1#: sense sequence 5′-GAACACUAUGGCUAUGCAUCC-3′, antisense sequence 5′-AUGCAUAGCCAUAGUGUUCUU-3′. siRNA 2#: sense sequence 5′-AUACUGGAAGUGCCUUAUUUATT-3′, antisense sequence 5′-UAAAUAAGGCACUUCCAGUAUTT-3′. siRNA 3#: sense sequence 5′-CCCGCCAACGUUCCAAUUAUATT-3′, antisense sequence 5′-UAUAAUUGGAACGUUGGCGGGTT-3′.

### 2.11. Molecular Docking Protocol for Manidipine—NFAT2 Interaction Analysis

Molecular docking simulations were performed using AutoDock Vina 1.1.2 to investigate the binding interactions between Manidipine (PubChem CID: 4008) and the NFAT2 protein (UniProt ID: O95644). Structural preparation of the target protein involved removal of crystallographic water molecules and non-essential ligands using PyMOL 2.4, followed by hydrogen atom addition to optimize protonation states. Protein and ligand topologies were converted to PDBQT format via AutoDock Tools 1.5.6 for docking parameterization. A cubic docking grid encompassing the binding pocket was defined with dimensions 96 × 118 × 126 Å^3^ (grid spacing: 1.00 Å), centered at Cartesian coordinates (−7.675, 3.754, −0.227). Following standardized protocols, the computational screening generated ten thermodynamically favorable binding poses. The predominant interaction mode was selected based on minimal computed binding energy (ΔG) and maximal cluster occupancy. Final binding geometries were reconstructed using PyMOL 2.4, employing advanced visualization techniques to analyze molecular interactions. The dissociation constant (Ki) was derived from docking scores using the thermodynamic relationship: $Ki=Kd=e^{\frac{\Delta G}{RT}}$

### 2.12. Drug Affinity Responsive Target Stability (DARTS) Experiment

For the drug-affinity responsive target stability (DARTS) assay, A549/T cells were mechanically homogenized in ice-cold 10 mM HEPES buffer supplemented with a protease-inhibitor cocktail. The homogenate was centrifuged at 14,000 rpm for 10 min at 4 °C to remove cellular debris, and the resulting supernatant was collected. Protein concentration was determined with the BCA assay. Aliquots containing equal amounts of protein were then incubated with manidipine for 1 h at 37 °C. Limited proteolysis was initiated by adding streptavidin protease at a protease/protein ratio of 1:2500 or 1:5000 at room temperature for 10 min. Digestion was terminated by the immediate addition of SDS loading buffer, followed by heating at 100 °C for 10 min. The samples were resolved by SDS-PAGE and subjected to immunoblotting for NFAT2 detection.

### 2.13. Statistical Analysis

All experiments were repeated at least three times, and the data were expressed as the mean ± SD unless noted otherwise. Data processing and analysis were performed using Microsoft Excel 2010 and GraphPad Prism 5.00. Statistical analysis involved Student’s *t*-test or one-way ANOVA, with significance defined as *p* < 0.05 (*), *p* < 0.01 (**), or *p* < 0.001 (***).

## 3. Results

### 3.1. Manidipine Enhances the Efficacy of PTX and DOX in A549/T and HCT-8/T Cells

To confirm drug resistance in the established resistant cell lines, we compared their sensitivity to paclitaxel (PTX) and doxorubicin (DOX) relative to their parental counterparts. The IC_50_ (Effective concentration 50%) values of PTX and DOX in A549/T cells were 92.19-fold and 13.14-fold higher, respectively, than in the drug-sensitive A549 cells, indicating successful induction of PTX resistance (Figure S1A,B). Similarly, the IC_50_ values of PTX and DOX in HCT-8/T cells were 201-fold and 17.15-fold higher, respectively, compared to the parental HCT-8 cells (Figure S1C,D). These results confirm that A549/T and HCT-8/T cells exhibit strong multidrug resistance.

**Figure 1:**
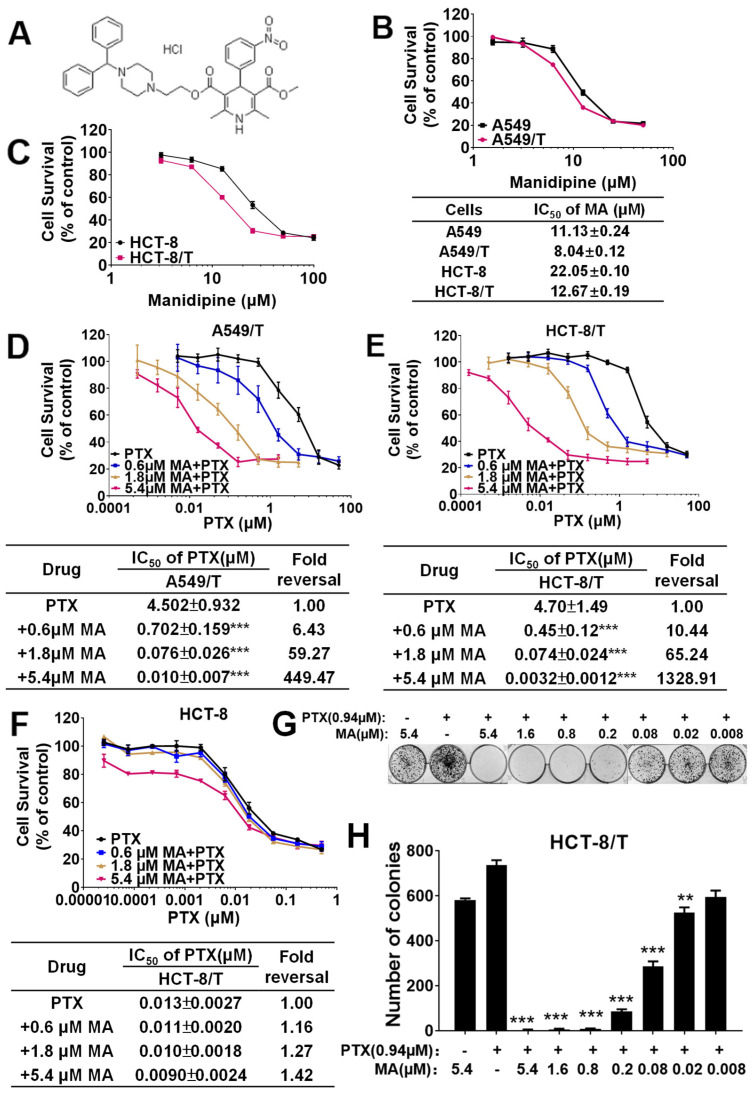
Manidipine (MA) overcomes paclitaxel (PTX) resistance in A549/T cells and HCT-8/T cells. (**A**) Chemical structure of MA. (**B**) Cytotoxicity of MA alone in A549 and A549/T cells. (**C**) Cytotoxicity of MA alone in HCT-8 and HCT-8/T cells. (**D**) MA reduces the IC_50_ of PTX in A549/T cells. (**E**) MA reduces the IC_50_ of PTX in HCT-8/T cells but not in drug-sensitive HCT-8 cells (**F**). Cells were treated with the indicated drugs for 48 h, and IC_50_ values were determined using the SRB assay. (**G**,**H**) Colony formation assay of PTX in the presence or absence of MA. Colony numbers were counted after Giemsa staining using the software of Quantity one-Colony counting. Data are presented as means ± SD from three independent experiments performed in triplicate. ** *p* < 0.01, *** *p* < 0.001, significantly different from control conditions without MA.

Next, we evaluated the intrinsic cytotoxicity of manidipine (Figure 1A) in both drug-sensitive and drug-resistant cell lines. The IC_50_ values of manidipine were 11.13 μM and 8.04 μM for A549 and A549/T cells, respectively (Figure 1B). In HCT-8 and HCT-8/T cells, the IC_50_ values were 22.05 μM and 12.67 μM, respectively (Figure 1C). To minimize potential cytotoxic effects of manidipine itself, we selected 5.4 μM as the maximum concentration for subsequent experiments, as this concentration exhibited less than 10% growth inhibition in both cell lines.

To determine whether manidipine reverses ABC transporter-mediated multidrug resistance (MDR), we examined its effect on PTX resistance in drug-resistant A549/T and HCT-8/T cells. In the ABCB1-overexpressing A549/T cell line, manidipine at 0.6, 1.8, and 5.4 μM reduced the IC_50_ of PTX by 6.43-, 59.27-, and 449.47-fold, respectively (Figure 1D). A similar reversal effect was observed in HCT-8/T cells, where manidipine at the same concentrations reduced the IC_50_ of PTX by 10.44-, 65.24-, and 1328.91-fold, respectively (Figure 1E).

In addition, manidipine enhanced the cytotoxicity of other P-gp substrate chemotherapeutics in HCT-8/T cells, including DOX (1.63-, 8.40-, and 18.05-fold), docetaxel (TXT) (5.12-, 41.66-, and 604-fold), and daunorubicin (DAU) (9.74-, 40.94-, and 136.33-fold) in a concentration-dependent manner. However, manidipine had little effect on the IC_50_ of 5-fluorouracil (5-FU), a non-P-gp substrate (Table 1). Furthermore, in drug-sensitive HCT-8 cells, manidipine did not significantly alter the IC_50_ of PTX (Figure 1F), suggesting that its MDR-reversing effect is specific to P-gp-overexpressing cells.

To further investigate the long-term effect of manidipine in reversing ABCB1-mediated MDR, we performed a colony formation assay. As shown in Figure 1G,H, complete inhibition of colony formation was achieved when 0.94 μM PTX was combined with manidipine at concentrations ≥0.2 μM. However, no significant inhibition was observed when cells were treated with 0.94 μM PTX or 5.4 μM manidipine alone. These results indicate that manidipine selectively enhances the cytotoxic effects of P-gp substrate drugs in a dose-dependent manner in ABCB1-overexpressing MDR cancer cells, supporting its potential as a potent MDR reversal agent in vitro.

### 3.2. Manidipine Combination with PTX to Induce Apoptosis in Drug-Resistant Cells

**Figure 2:**
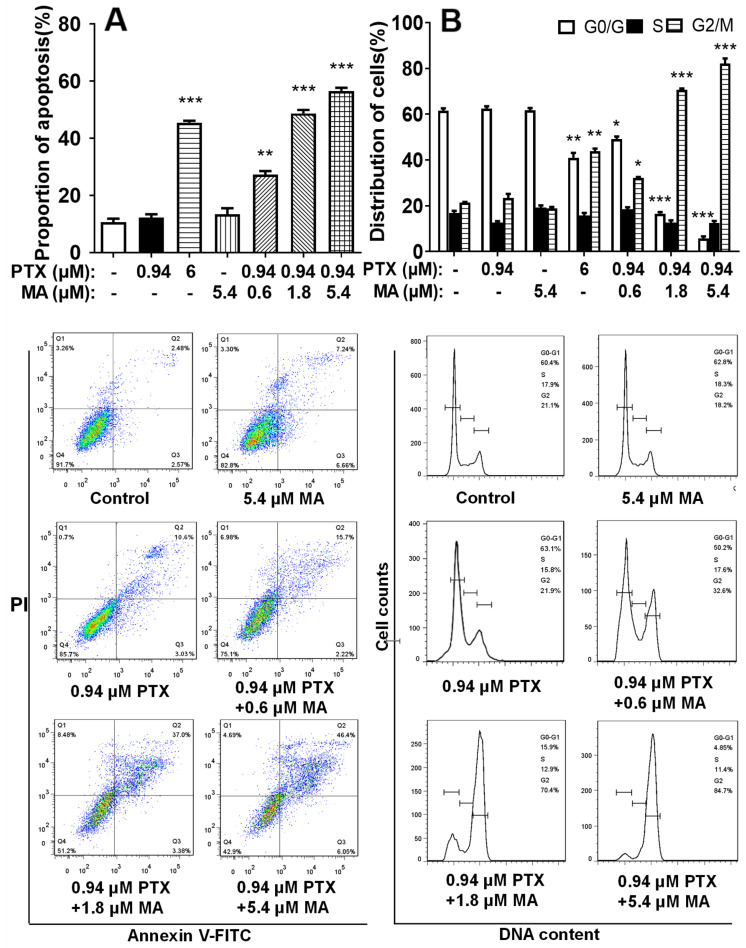
Effects of the combination of manidipine (MA) and PTX (0.94 μM) on the apoptosis and cell cycle. (**A**) Cells were treated with varying concentrations of MA and PTX (0.94 μM) for 48 h, stained with Annexin V-FITC, and analyzed by flow cytometry. (**B**) Cell cycle distribution was assessed by flow cytometry following treatment with MA and PTX. Data represent the mean ± SD (n = 3). * *p* < 0.05, ** *p* < 0.01, *** *p* < 0.001 vs. control.

To investigate the effect of manidipine on PTX-induced apoptosis in HCT-8/T cells, we performed flow cytometric analysis. The results showed that the combination of manidipine (0.6–5.4 μM) and PTX (0.94 μM) significantly enhanced apoptosis in a dose-dependent manner (Figure 2A). In contrast, neither 5.4 μM manidipine nor 0.94 μM PTX alone induced significant apoptosis. Notably, even at 0.6 μM, manidipine potentiated PTX-induced apoptosis to a level comparable to that observed with 6 μM PTX (the IC_50_ of PTX).

Next, we assessed whether the synergistic effect of manidipine and PTX influenced cell cycle progression in HCT-8/T cells using flow cytometry. PTX treatment alone resulted in 61.7% of cells in the G0/G1 phase, 16.5% in the S phase, and 21.3% in the G2/M phase (Figure 2B). However, when cells were co-treated with 5.4 μM manidipine and 0.94 μM PTX, the distribution shifted markedly, with 5.53% of cells in the G0/G1 phase, 12.7% in the S phase, and 81.19% in the G2/M phase. Notably, treatment with either 5.4 μM manidipine or 0.94 μM PTX alone had no significant effect on cell cycle distribution, suggesting that manidipine synergizes with PTX to induce G2/M arrest in drug-resistant cells.

### 3.3. Manidipine Exerts Synergistic Effect with PTX in MDR Cells

To quantitatively evaluate the synergistic effect between manidipine and PTX, we performed combination index (CI) analysis (Figure S2). The CI values at 50% (ED_50_) and 90% (ED_90_) of cell inhibition were 1.96 × 10^−5^ and 6.9 × 10^−13^, respectively, indicating a very strong synergistic cytotoxic effect (CI < 0.1) in HCT-8/T cells (Table 2).

Using the CalcuSyn simulation, the ED_50_ values for manidipine and PTX alone in HCT-8/T cells were 14.8226 μM and 5.36072 μM, respectively. However, when combined with 1.47 × 10^−4^ μM manidipine, the ED_50_ of PTX decreased dramatically to 5.16 × 10^−5^ μM, representing a 103,889.92-fold reduction. These findings confirm that manidipine strongly enhances the cytotoxicity of PTX in MDR cancer cells.

### 3.4. Manidipine Activates the P-gp ATPase Activity

**Figure 3:**
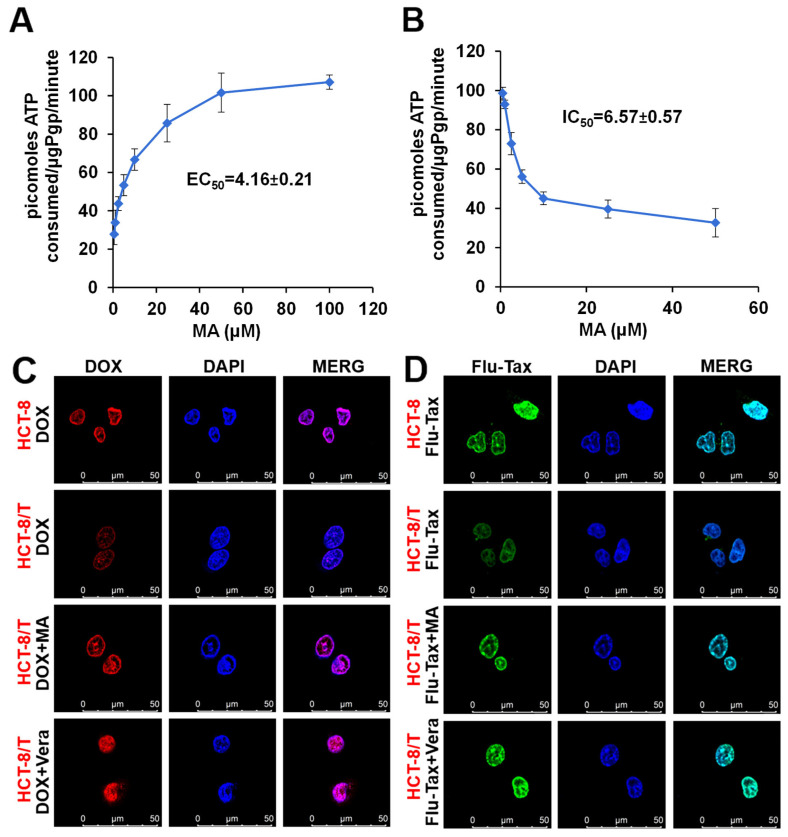
Manidipine (MA) inhibits the drug efflux transport P-gp. (**A**) EC_50_ measurement for MA-stimulated P-gp ATPase activity. (**B**) IC_50_ measurement for MA inhibition of 200 μM verapamil-stimulated P-gp ATPase activity. Luminescence was read on a luminometer and data was analyzed as described in Section 2. HCT-8 cells or HCT-8/T Cells treated with 5 μM DOX (**C**) or 2 μM Flu-Tax (**D**) in the absence or presence of 5.4 μM MA, or 50 μM Vera (verapamil, positive control) as indicated. Intracellular DOX and Flu-Tax accumulation were observed with a florescence microscope. The experiments were repeated for at least 3 times, presented are representative images.

Since the efflux function of ABCB1 (P-gp) is closely linked to ATP hydrolysis, we measured P-gp ATPase activity in the presence of different concentrations of manidipine. As shown in Figure 3A, manidipine stimulated ATPase activity in a dose-dependent manner, with an EC_50_ (Effective concentration 50%) of 4.16 μM and a maximum stimulation of 2-fold the basal activity. These findings suggest that manidipine may act as a substrate for P-gp by interacting with its drug-substrate binding site.

### 3.5. Manidipine Increases the Intracellular Accumulation of DOX and Flu-Tax

The findings presented above demonstrate that manidipine significantly reverses multidrug resistance (MDR) in HCT-8/T and A549/T cell lines when exposed to chemotherapeutic agents such as paclitaxel (PTX). However, the precise mechanisms underlying this effect remain unclear. To elucidate these mechanisms, we evaluated the influence of manidipine on the intracellular accumulation of doxorubicin (DOX) and Flu-Tax, a fluorescent taxol derivative, in both drug-sensitive HCT-8 cells and their MDR counterparts (HCT-8/T cells) using fluorescence microscopy and flow cytometry.

In the absence of manidipine, the fluorescence intensity of DOX and Flu-Tax was markedly higher in drug-sensitive parental cells compared to MDR cells (Figure 3C,D). Notably, treatment with manidipine (5.4 µM) significantly enhanced the intracellular accumulation of DOX (Figure 3C) and Flu-Tax (Figure 3D) in MDR cells, comparable to the effect of the positive control verapamil (50 µM), while having no significant impact on drug-sensitive HCT-8 cells. These results suggest that manidipine effectively increases the intracellular retention of chemotherapeutic agents in MDR cells, thereby enhancing their cytotoxic effects.

### 3.6. Manidipine in Combination with PTX Inhibits the Growth of A549/T Xenograft in Nude Mice

**Figure 4:**
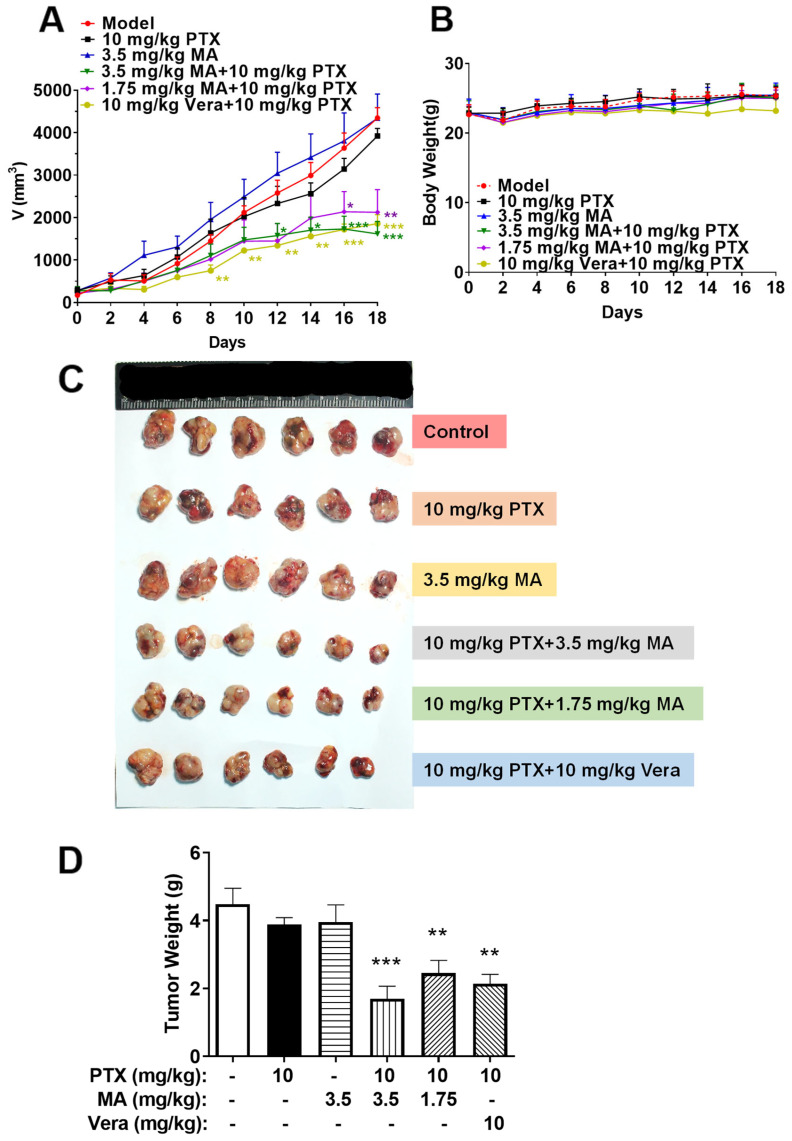
Manidipine (MA) enhances the anti-cancer activity of paclitaxel (PTX) in drug-resistant tumors. (**A**) the change in the tumor volume. (**B**) Changes in mouse body weight during resistance to cancer formation. (**C**) Tumor specimens were photographed under standardized conditions. (**D**) the change in the tumor weight in the presence or absence of MA. * *p* < 0.05, ** *p* < 0.01, *** *p* < 0.001, significantly different from those obtained in the absence of MA.

To further validate our findings, we established an in vivo A549/T xenograft model in BALB/c nude mice to assess the potential of manidipine in overcoming PTX resistance. As shown in Figure 4, tumor growth did not differ significantly among the groups treated with saline, manidipine, or PTX alone. However, co-administration of manidipine and PTX significantly suppressed tumor growth (Figure 4A), suggesting that manidipine effectively reverses ABCB1-mediated MDR in vivo.

Moreover, the body weight of the mice remained stable across all groups (Figure 4B), indicating that the combination treatment did not affect the overall growth situation. Notably, tumor growth inhibition in the manidipine-PTX combination group was superior to that observed in the verapamil-PTX group (Figure 4C,D), further supporting the efficacy of manidipine as an MDR reversal agent. These findings highlight the potential of manidipine as an adjunct to conventional chemotherapy, enhancing PTX sensitivity and offering a promising strategy for overcoming MDR in cancer therapy.

### 3.7. Manidipine Inhibits Drug Efflux by Mitigating Ca^2+^ Influx

**Figure 5:**
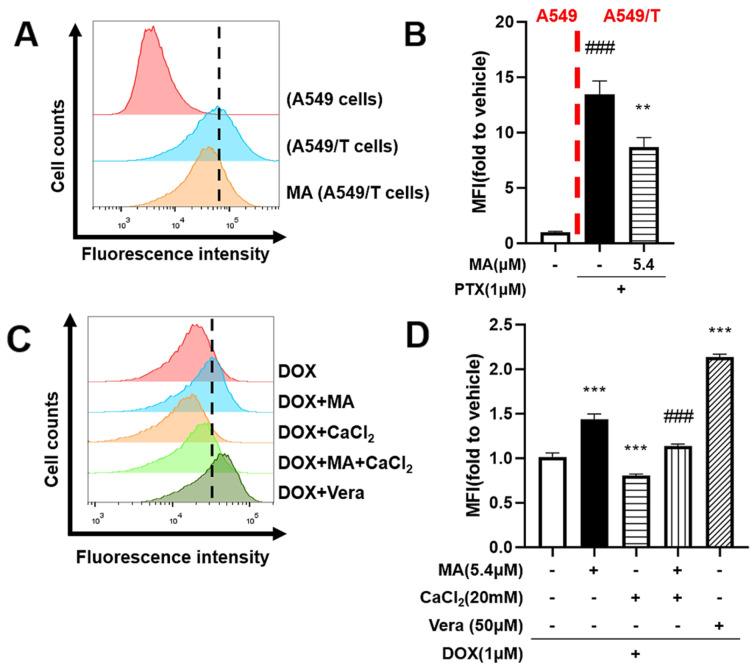
Manidipine (MA) inhibits the drug efflux via modulating calcium. (**A**,**B**) Calcium assay in A549 and A549/T cells. Effect of MA on calcium concentration in A549 and A549/T cells treated with Fluo-4 AM in the absence or presence of 5.4 μM MA. Intracellular DOX was evaluated by measuring fluorescence with flow cytometry. The experiments were repeated at least 3 times, presented are representative images. The data were expressed as mean ± SD (n = 3), *** *p* < 0.001 vs. A549, ^###^ *p* < 0.001 vs. A549/T. (**C**,**D**) Effect of MA on intracellular accumulation of doxorubicin (DOX) in A549/T cells treated with 1μM DOX for 4 h in the absence or presence of 5.4μM MA and 20 mM CaCl_2_, and 50μM Vera (verapamil, positive control) as indicated. Intracellular DOX evaluated by measuring fluorescence with flow cytometry. The experiments were repeated for at least 3 times, presented are representative images. The data were expressed as mean ± SD (n = 3), ** *p* < 0.01, *** *p* < 0.001 vs. A549/T, ^###^ *p* < 0.001 vs. MA group. MFI, Mean fluorescence intensity.

To determine whether the MDR reversal effect of manidipine is linked to calcium ion regulation, we utilized a calcium-sensitive fluorescent probe to assess intracellular calcium concentrations in A549 and A549/T cells. A significant elevation in calcium levels was observed in drug-resistant cells compared to their drug-sensitive counterparts. Notably, treatment with manidipine markedly reduced intracellular calcium levels in A549/T cells (Figure 5A,B).

Further investigation revealed that increasing intracellular calcium levels with CaCl_2_ antagonized the sensitizing effect of manidipine on DOX retention in drug-resistant cells (Figure 5C,D). Importantly, elevated calcium ion levels were correlated with increased drug efflux, reinforcing the link between calcium signaling and drug expulsion mechanisms. Collectively, these findings indicate that manidipine reverses MDR by modulating calcium ion homeostasis, thereby reducing drug efflux and enhancing intracellular drug retention.

### 3.8. NFAT2 Was Associated with Drug Resistance

**Figure 6:**
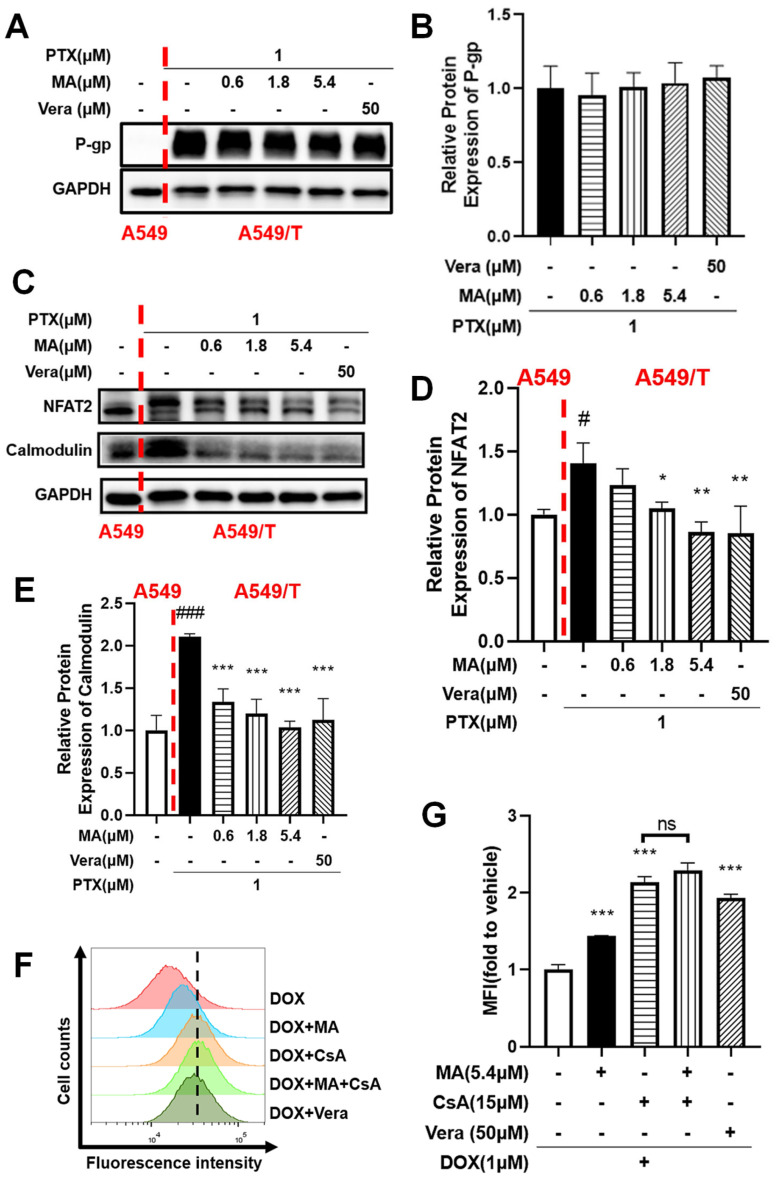
Manidipine (MA) modulates the calcium signaling and NFAT2 pathways. (**A**–**E**) Effect of MA on NFAT2 and Calmodulin expression via Western blot. A549/T cells or A549 cells were treated with MA at various concentrations for 48 h, and 50μM Vera (verapamil, positive control) as indicated. The experiments were performed three times. The data were expressed as mean ± SD (n = 3), ^#^ *p* < 0.05, ^###^ *p* < 0.001 vs. A549 cells, * *p* < 0.05, ** *p* < 0.01, *** *p* < 0.001 vs. A549/T cells. (**F**,**G**) Effect of MA on intracellular accumulation of doxorubicin (DOX) in A549/T cells treated with 1μM DOX for 4 h in the absence or presence of 5.4μM MA and 15μM CsA (an NFAT inhibitor), and 50μM Vera (verapamil, positive control) as indicated. Intracellular DOX evaluated by measuring fluorescence with flow cytometry. The experiments were repeated for at least 3 times, presented are representative images. The data were expressed as mean ± SD (n = 3), *** *p* < 0.001 vs. DOX group. The abbreviation "ns" stands for "not significant" in statistical contexts.

To further explore the molecular basis of the MDR-reversal effect of manidipine, we examined its influence on P-glycoprotein (P-gp, encoded by ABCB1) expression at the protein level. Within the therapeutic concentration range (0.6–5.4 µM), manidipine did not significantly alter P-gp expression in A549/T cells (Figure 6A,B), indicating that its MDR reversal effect is not due to suppression of ABCB1 expression. Instead, these findings suggest that manidipine enhances intracellular drug accumulation by inhibiting P-gp transporter activity.

Next, we investigated the role of nuclear factor of activated T cells 2 (NFAT2) in MDR. Western blot analysis revealed that NFAT2 expression was significantly upregulated in A549/T cells compared to A549 cells (Figure 6C–E). Treatment with manidipine (5.4 µM) downregulated NFAT2 expression in a dose-dependent manner, and the effect was also observed with verapamil. Additionally, calmodulin, a key regulator of NFAT2 activity, was highly expressed in drug-resistant tumor cells, and manidipine treatment led to its significant downregulation.

To further elucidate the mechanism by which manidipine reverses MDR, we evaluated its effect on intracellular drug accumulation using flow cytometry. As shown in Figure 6F,G, treatment with manidipine (5.4 µM) significantly increased the intracellular accumulation of doxorubicin (DOX) in A549/T cells, comparable to the effect of the positive control verapamil (50 µM). To assess the involvement of the NFAT pathway in this process, we examined the impact of cyclosporine A (CsA), a well-established NFAT inhibitor [26,30,31]. Treatment with CsA significantly increased intracellular DOX accumulation in A549/T cells to the same maximal level observed with manidipine alone. Co-administration of CsA and manidipine did not produce any further increase, indicating that both agents operate on the same NFAT-dependent efflux pathway. Once NFAT-mediated signaling is fully suppressed by CsA, manidipine is unable to exert additional effects, supporting the conclusion that the chemosensitisation conferred by manidipine is exerted—at least in large part—through NFAT inhibition.

These findings highlight the critical role of NFAT in regulating intracellular drug efflux and underscore its involvement in the chemosensitizing effect of manidipine.

### 3.9. NFAT2 Silencing Overcomes Drug Efflux

**Figure 7:**
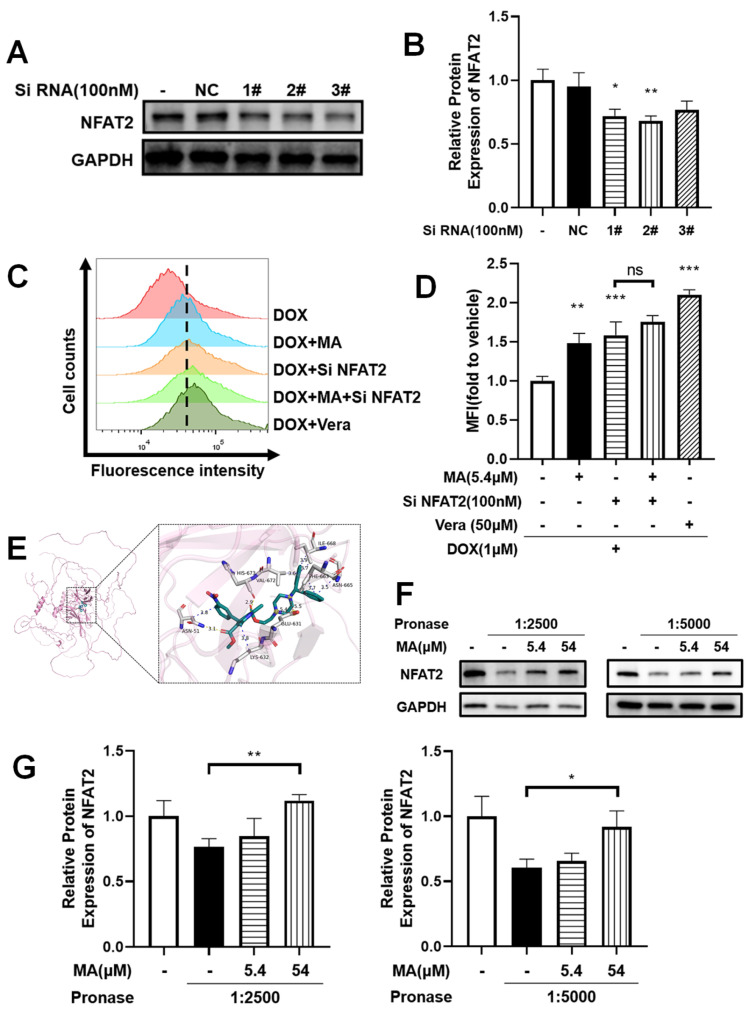
Manidipine (MA) overcomes drug efflux by modulating the NFAT2 pathways. (**A**,**B**) NFAT2 expression in A549/T cells was knocked down by siRNA. The experiments were repeated for at least 3 times, presented are representative images. The data were expressed as mean ± SD (n = 3), * *p* < 0.05, ** *p* < 0.01 vs. NC group. (**C**,**D**) Effect of MA on intracellular accumulation of DOX in A549/T cells treated with 1μM DOX for 4 h in the absence or presence of 5.4 μM MA and siRNA, and 50 μM Vera (verapamil, positive control) as indicated. Intracellular DOX evaluated by measuring fluorescence with flow cytometry. The experiments were repeated for at least 3 times, presented are representative images. The data were expressed as mean ± SD (n = 3), ** *p* < 0.01, *** *p* < 0.001 vs. DOX group. (**E**) Molecular docking studies revealed the direct interaction between MA and NFAT2, highlighting the key residues involved in the binding. (**F**,**G**) DARTS analysis identified the interaction between MA and NFAT2 in A549/T cells (n = 3). The experiments were repeated for at least 3 times, presented are representative images. The data were expressed as mean ± SD (n = 3).

To further validate the role of NFAT2 in MDR, we examined whether NFAT2 knockdown could counteract drug efflux in A549/T cells. Small interfering RNA (siRNA) was used to suppress NFAT2 expression (Figure 7A,B), and based on preliminary screening, siRNA #2 was selected for subsequent experiments. NFAT2 knockdown resulted in a significant increase in intracellular DOX accumulation (Figure 7C,D). Notably, co-treatment with manidipine did not further enhance DOX retention, reinforcing the notion that NFAT2 is a key mediator of manidipine’s effect on intracellular drug accumulation.

Together, these findings demonstrate that manidipine effectively increases intracellular drug retention in MDR cells by targeting NFAT2, thereby enhancing chemosensitivity. This study provides novel insights into the role of NFAT in drug resistance and suggests that its inhibition may represent a promising strategy for overcoming MDR in cancer therapy.

### 3.10. Manidipine Specifically Targets NFAT2 Through Direct Molecular Interaction

Molecular docking analysis revealed binding parameters between manidipine and the NFAT2 protein derived from three independent computational analyses. The calculated binding energies yielded average values of −30.5567 kJ/mol, with a corresponding mean predicted binding affinity of 4.3967 μM. Computational modeling demonstrated that manidipine formed stable molecular interactions with NFAT2, as evidenced by direct binding patterns visualized in the simulated complexes (Figure 7E).

### 3.11. Protection of NFAT2 from Proteolysis by Manidipine

To explore the nature of this interaction, the drug affinity responsive target stability (DARTS) assay was employed to detect the ligand-protein interactions [32]. Incubating MA with lysates of A549/T cells, followed by digestion with streptavidin protease at a pronase: protein ratio of 1:2500 and 1: 5000. The subsequent immunoblotting analysis showed that MA effectively protected the NFAT2 protein from proteolysis (Figure 7F,G), thereby indicating that MA is a ligand of NFAT2.

## 4. Discussion

In this study, we demonstrated that manidipine significantly reverses drug resistance, as evidenced by its ability to enhance paclitaxel (PTX) chemosensitivity, restore cell cycle arrest, and inhibit drug efflux. These effects were further validated in vivo. Mechanistically, our findings reveal that manidipine reduces drug efflux activity in resistant cancer cells by modulating intracellular calcium concentrations and targeting nuclear factor of activated T cells 2 (NFAT2), ultimately re-sensitizing these cells to chemotherapy. Although our functional and biochemical data point to P-gp as the primary target of manidipine, we have not formally excluded contributions from MRP-1 or ABCG2. Future work will profile the expression of these transporters as well as their contribution to the effects of manidipine on paclitaxel chemosensitivity.

Our findings align with a growing body of research demonstrating the role of calcium channel blockers (CCBs) in overcoming multidrug resistance (MDR) in cancer. Previous studies have shown that verapamil [11], diltiazem, and nifedipine [33], can enhance chemosensitivity by inhibiting P-glycoprotein (P-gp) function, thereby increasing intracellular drug accumulation. Moreover, although manidipine stimulates P-gp ATPase activity and competes with verapamil-stimulated hydrolysis, our current functional assays cannot resolve whether it contacts the ATPase site, the drug-binding pocket, or both. Until high-resolution structural or mutagenesis data are available, we simply note that its behavior is consistent with that of a classical substrate that can engage the transporter’s catalytic cycle. However, our study uniquely highlights a dual mechanism of action for manidipine: not only does it inhibit drug efflux via P-gp modulation, but it also targets NFAT2, a key regulator of MDR. This novel insight provides a more comprehensive understanding of how CCBs can be leveraged to counteract MDR [24].

The involvement of NFAT2 in cancer progression and drug resistance has been well documented. NFAT2 has been implicated in promoting metastasis, tumorigenesis, and proliferation, as well as shaping the tumor microenvironment [24]. While previous research has primarily focused on NFAT2’s role in immune regulation and cancer progression, our study provides new evidence that NFAT2 directly contributes to drug resistance by modulating intracellular drug retention. These findings further support the notion that NFAT2 may serve as a potential therapeutic target for MDR reversal.

Despite the promising results, our study has several limitations. First, the in vivo experiments were conducted using a limited number of animal models, which may not fully capture the complexity and heterogeneity of human cancer. Expanding these studies to include additional preclinical models, such as patient-derived xenografts (PDX) or genetically engineered mouse models (GEMMs), would provide a more robust assessment of the efficacy of manidipine. Second, although we observed no significant adverse effects in the animal models, the potential for systemic toxicity in humans remains unknown. Fortunately, manidipine was reported well well-tolerated in clinical trials for anti-hypertension [14]. And HPLC/ESIMS can be used to determine the pharmacokinetic parameters of manidipine [34], all of which laid the foundation for the further development and clinical application of manidipine in the future. Clinically, after oral administration of 20 mg of manidipine, the average maximum plasma concentration of manidipine was (7.8 ng/mL), and the plasma protein binding rate of this drug could reach 99%, and it was widely distributed in tissues. Moreover, in patients with essential hypertension, the blood-pressure-lowering effect achieved with manidipine 10 or 20 mg once daily is comparable to that of other calcium-channel antagonists such as felodipine, nicardipine and nifedipine [13]. In terms of drug metabolism, manidipine is the major substrate of CYP3A4 [35]. Co-administration of strong CYP3A4 inhibitors (ketoconazole) is expected to raise manidipine exposure, whereas dexamethasone (standard paclitaxel pre-medication) or inducers (phenytoin) may lower it below the efficacious threshold [36,37,38].

Given that calcium channel blockers are widely used for hypertension treatment, their safety profile in non-cancerous tissues must be carefully evaluated in the context of long-term cancer therapy. Future studies should focus on pharmacokinetics, biodistribution, and potential cardiotoxicity or neurotoxicity associated with prolonged manidipine treatment.

Additionally, while our study demonstrated that manidipine effectively reverses MDR in A549/T cells, the possibility of cancer cells developing resistance to manidipine itself cannot be ruled out. Long-term studies investigating adaptive resistance mechanisms and potential compensatory pathways should be conducted to assess the durability of MDR-reversing effects by manidipine.

Future research should aim to validate the clinical efficacy and safety of manidipine in overcoming MDR by expanding studies to a broader range of cancer types and more diverse patient populations. Investigating the potential synergistic effects of manidipine in combination with other chemotherapeutic agents or targeted therapies could provide valuable insights into optimizing treatment regimens.

Moreover, a deeper understanding of the molecular mechanisms by which manidipine modulates NFAT2 and calcium signaling pathways in cancer cells could pave the way for the development of more selective NFAT2 inhibitors or novel CCB-based therapies specifically designed for MDR reversal. Identifying predictive biomarkers for patient stratification may also help tailor treatments for individuals most likely to benefit from NFAT2-targeted strategies.

## 5. Conclusions

Overall, our study provides compelling evidence that manidipine reverses MDR by targeting NFAT2 and calcium signaling, leading to increased intracellular drug retention and enhanced chemosensitivity. These findings offer new insights into the molecular basis of MDR and highlight the potential of repurposing calcium channel blockers as MDR-reversal agents in cancer therapy. Future studies focusing on clinical translation, safety profiling, and combinational therapeutic strategies will be essential to fully realize the potential of manidipine in overcoming drug resistance.

## Figures and Tables

**Table 1:** Manidipine reverses P-gp-mediated drug resistance to chemotherapeutic agents in HCT-8/T cells.

Drug	IC_50_ (μM)	Fold Reversal
	HCT-8/T	
PTX	4.70 ± 1.49	1
+0.6 μM MA	0.45 ± 0.12 ***	10.44
+1.8 μM MA	0.074 ± 0.024 ***	65.24
+5.4 μM MA	0.0032 ± 0.0012 ***	1328.91
DOX	4.26 ± 1.11	1.00
+0.6 μM MA	2.62 ± 1.12	1.63
+1.8 μM MA	0.51 ± 0.16	8.40
+5.4 μM MA	0.24 ± 0.067 ***	18.05
TXT	1.21 ± 0.22	1.00
+0.6 μM MA	0.24 ± 0.030 **	5.12
+1.8 μM MA	0.029 ± 0.0053 ***	41.66
+5.4 μM MA	0.0023 ± 0.0014 ***	604
DAU	2.045 ± 0.43	1.00
+0.6 μM MA	0.210 ± 0.062 ***	9.74
+1.8 μM MA	0.053 ± 0.012 ***	40.94
+5.4 μM MA	0.015 ± 0.0061 ***	136.33
5-FU	134.62 ± 21.74	1.00
+0.6 μM MA	127.33 ± 28.83	1.057
+1.8 μM MA	204.00 ± 15.62	0.66
+5.4 μM MA	264.73 ± 32	0.508

IC_50_ values are represented as means ± SD of three independent experiments performed in triplicate. ** *p* < 0.01, *** *p* < 0.001, significantly different from those obtained in the absence of MA.

**Table 2:** Synergistic effect of manidipine and PTX based on CI values.

Data for Fa = 0.5	CI Value	Dose MA (µM)	Dose PTX (µM)
Manidipine	/	14.8226	/
PTX	/	/	5.36072
Manidipine + PTX	1.96 × 10^−5^	1.47 × 10^−4^	5.16 × 10^−5^
Data for Fa = 0.9	CI value	Dose MA (µM)	Dose PTX (µM)
Manidipine	/	3.30204	/
PTX	/	/	0.85596
Manidipine + PTX	6.9 × 10^−13^	9.6 × 10^−13^	3.4 × 10^−13^

CI values: synergism (0.3–0.7), strong synergism (0.1–0.3), very strong synergism (CI < 0.1). Note: CI values < 1 indicate a synergistic effect, CI = 1 represents an additive effect, and CI > 1 indicates antagonism. PTX sensitivity was significantly enhanced in manidipine-treated A549/T cells.

## Data Availability

The datasets used and/or analyzed during the current study are available from the corresponding author on reasonable request.

## Supplementary Materials

The following supporting information can be downloaded at: https://www.mdpi.com/article/10.3390/cancers17203289/s1, Figure S1. Multidrug resistance in drug-resistant cells (A549/T, HCT-8/T) compared to their parental counterparts (A549, HCT-8). Figure S2. Quantitative diagnostic graphics for the synergistic effect between manidipine (MA) and PTX, generated via computer simulation. Figure S3. The original image Western blotting of Figure 6A; Figure S4: The original image Western blotting of Figure 6C; Figure S5. The original image Western blotting of Figure 7A; Figure S6. The original image Western blotting of Figure 7F.
