# Supplementary material for: Targeting NFAT2 for Reversing the P-gp-Mediated Multidrug Resistance to Paclitaxel by Manidipine
Source: Cancers (Basel). 2025 Oct 10;17(20):3289. doi: 10.3390/cancers17203289 (PMC12562668; doi:10.3390/cancers17203289)
Supplement: Supplementary file 1 [file cancers-17-03289-s001.zip › FigureS1-2.pdf]

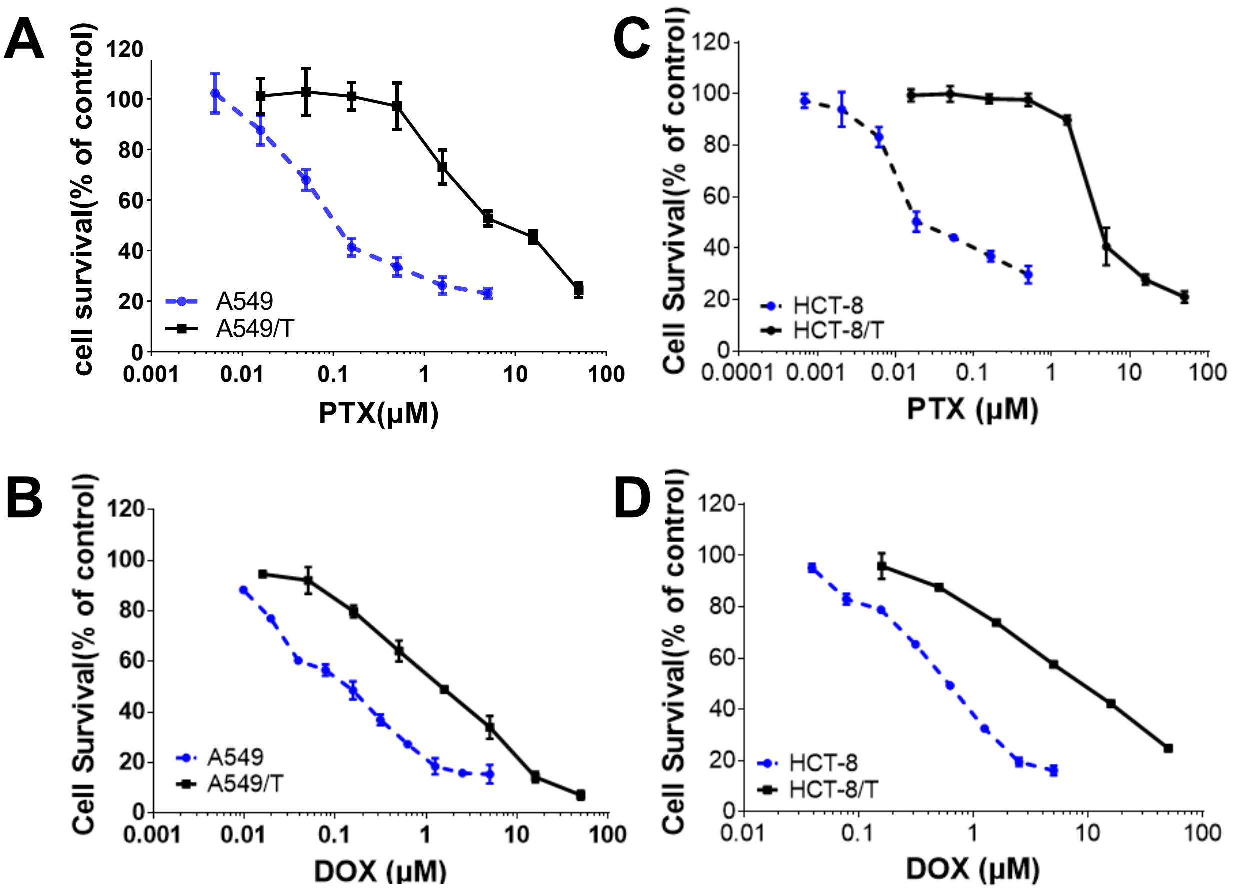

Drug	IC_{50}(μM)		Fold reversal
	A549/T	A549	
PTX	3.95±1.68***	0.04±0.02	92.19
DOX	1.38±0.44***	0.11±0.03	13.14

Drug	IC_{50}(μM)		Fold reversal
	HCT-8/T	HCT-8	
PTX	4.02±0.64***	0.02±0.004	201
DOX	8.92±0.55***	0.52±0.09	17.15

Figure S1. Multidrug resistance in drug-resistant cells (A549/T, HCT-8/T) compared to their parental counterparts (A549, HCT-8).

Cells were treated with various concentrations of PTX (A, C) and DOX (B, D) for 48 hours, and was assessed using the SRB assay.

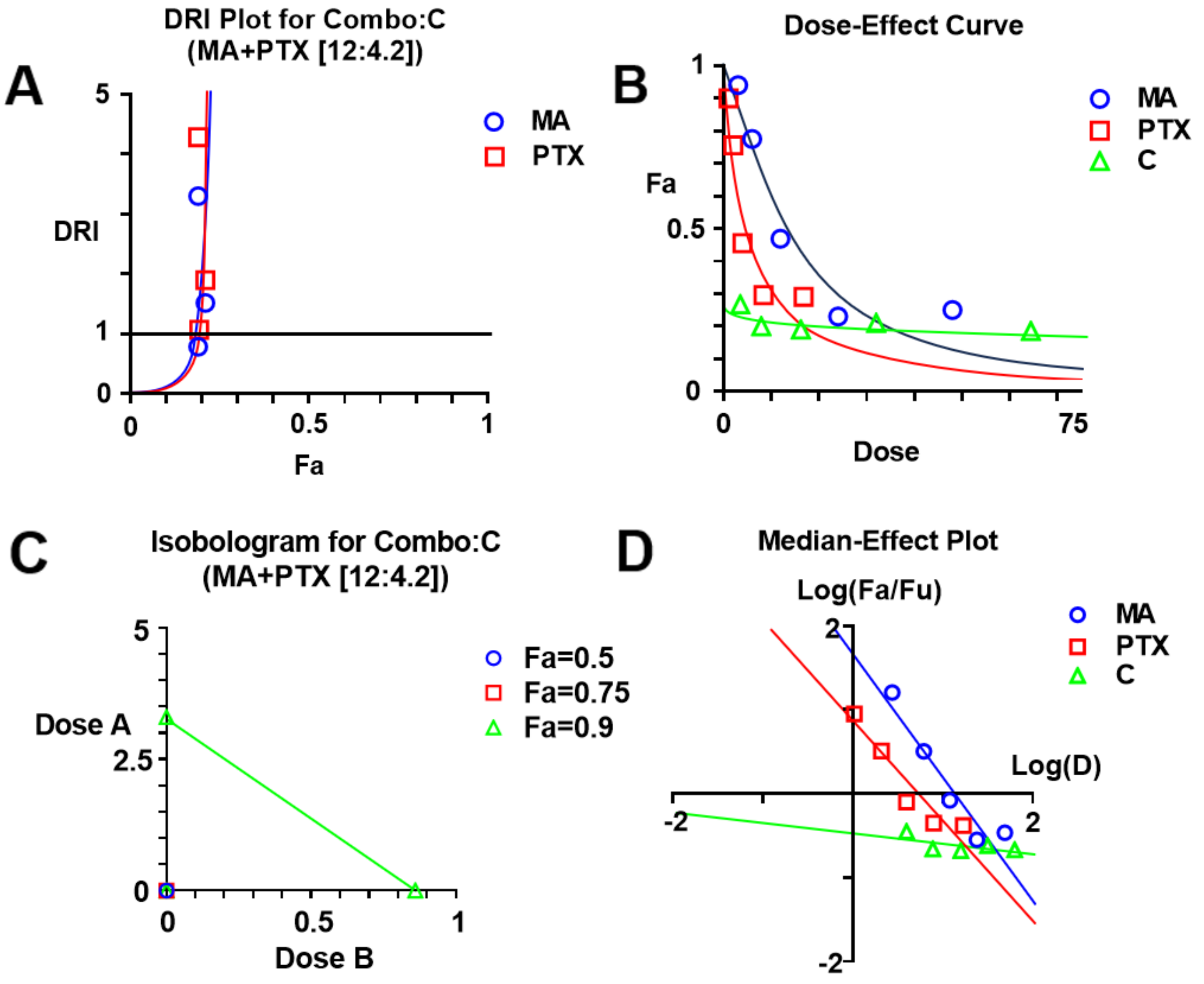

Figure S2. Quantitative diagnostic graphics for the synergistic effect between manidipine (MA) and PTX, generated via computer simulation.

(A) Fa-DRI plot (Chou-Martin plot) for constant-ratio combination analysis. (B) Fraction affected (Fa)-Dose plot for MA, PTX, and the combination. (C) Classic isobologram. (D) Fa-CI plot (Chou-Talalay plot).
