# Supplementary material for: Targeting NFAT2 for Reversing the P-gp-Mediated Multidrug Resistance to Paclitaxel by Manidipine
Source: Cancers (Basel). 2025 Oct 10;17(20):3289. doi: 10.3390/cancers17203289 (PMC12562668; doi:10.3390/cancers17203289)
Supplement: Supplementary file 1 [file cancers-17-03289-s001.zip › FigureS3-S6.pdf]

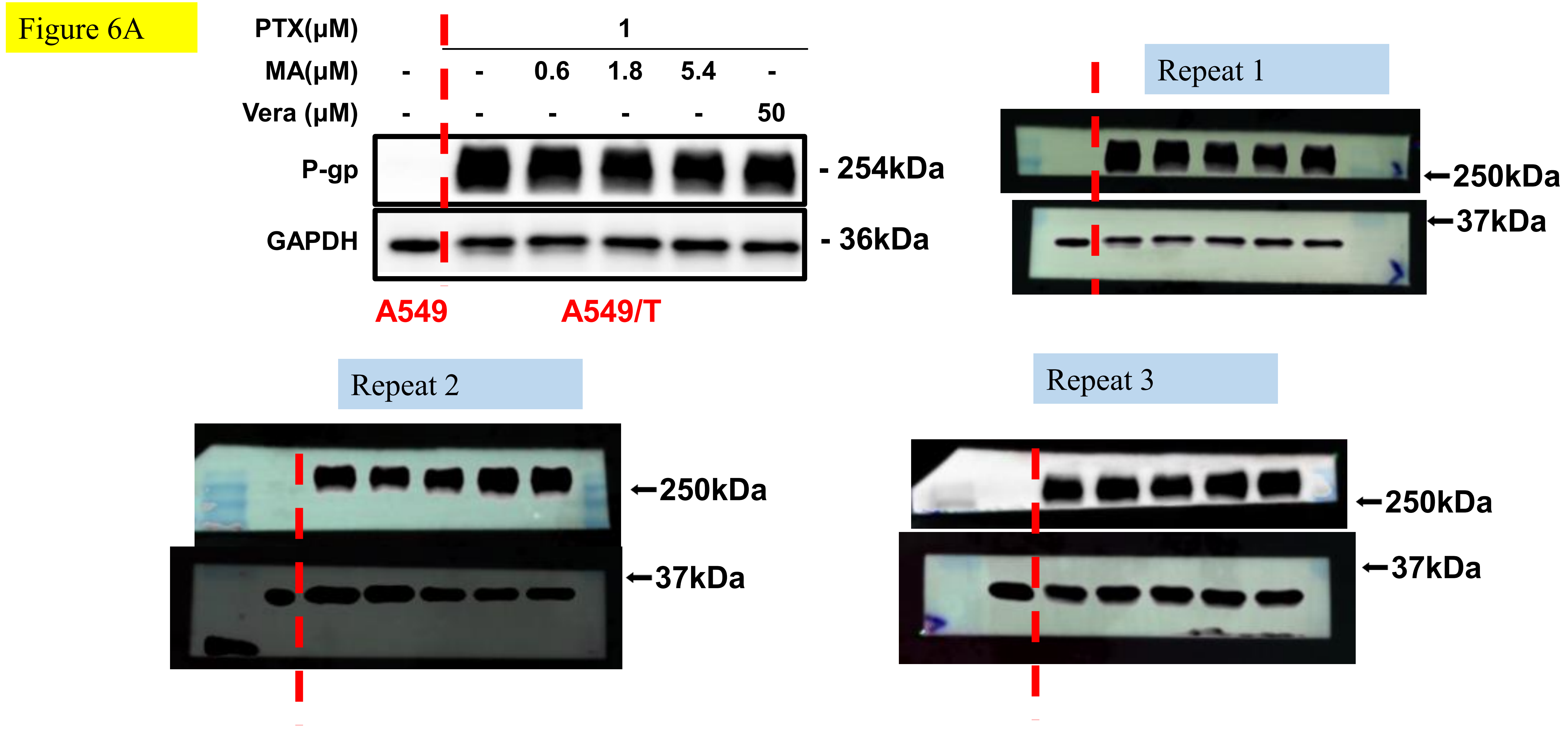

Figure S3. The original image Western blotting of Figure 6A

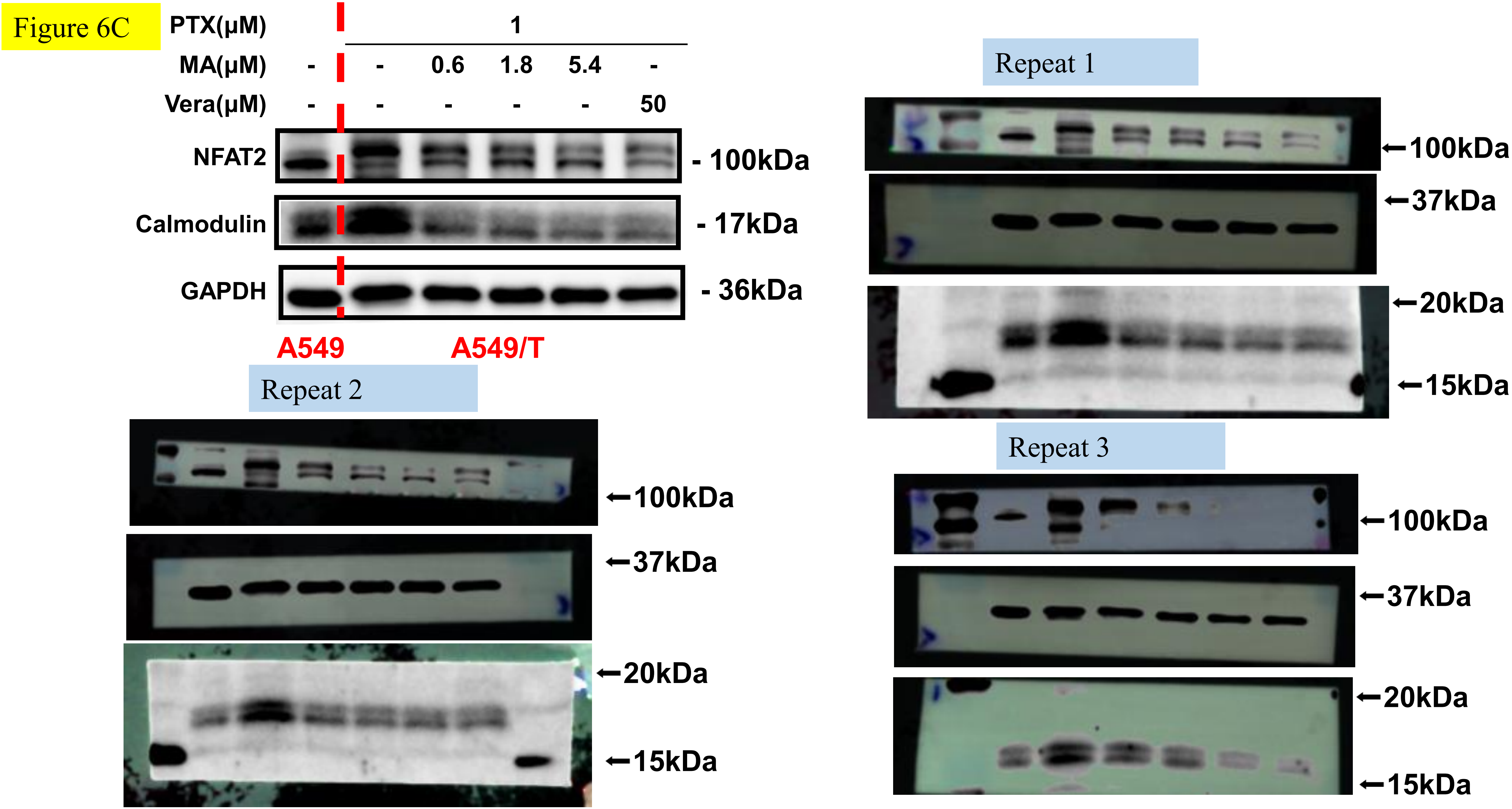

Figure S4. The original image Western blotting of Figure 6C

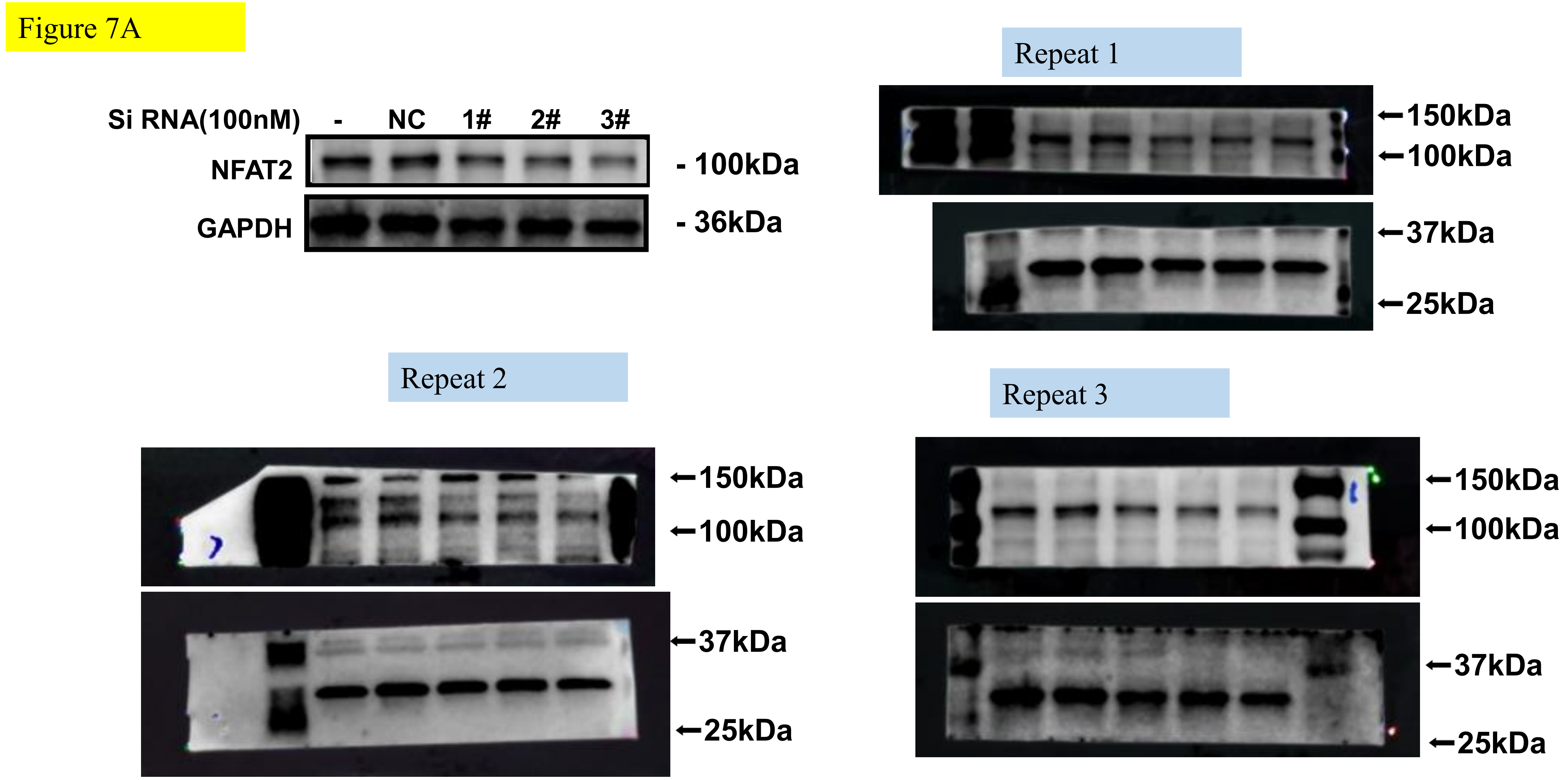

Figure S5. The original image Western blotting of Figure 7A

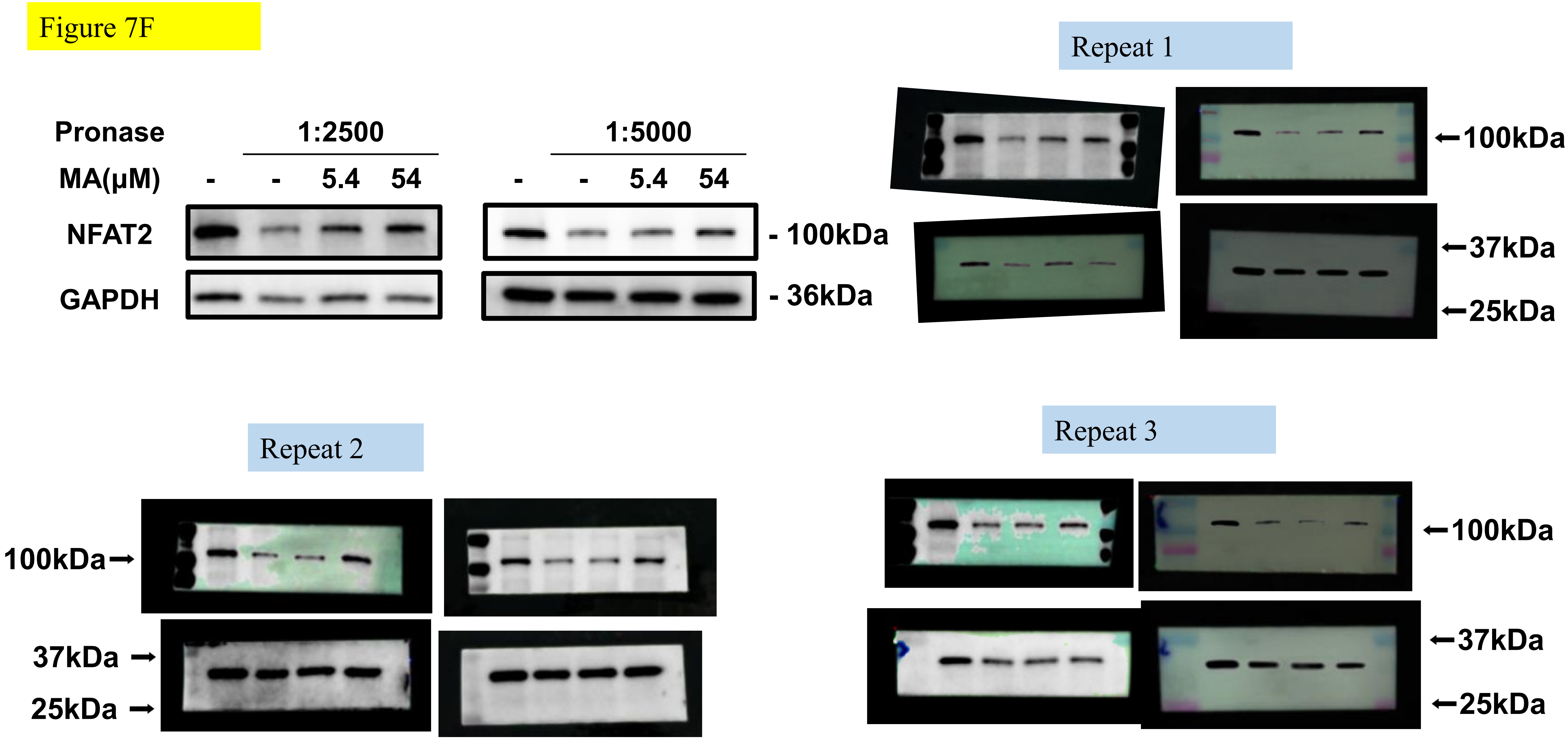

Figure S6. The original image Western blotting of Figure 7F
